# Prevalence of Depression in Older Nursing Home Residents in High and Low Altitude Regions: A Comparative Study

**DOI:** 10.3389/fpsyt.2021.669234

**Published:** 2021-06-22

**Authors:** Fei Wang, Shou Liu, Qinge Zhang, Chee H. Ng, Xiling Cui, Dexing Zhang, Yu-Tao Xiang

**Affiliations:** ^1^Guangdong Mental Health Center, Guangdong Academy of Medical Sciences, Guangdong Provincial People's Hospital, Guangzhou, China; ^2^Department of Public Health, Medical College, Qinghai University, Xining, China; ^3^The National Clinical Research Center for Mental Disorders and Beijing Key Laboratory of Mental Disorders, Beijing Anding Hospital and the Advanced Innovation Center for Human Brain Protection, Capital Medical University, Beijing, China; ^4^Department of Psychiatry, The Melbourne Clinic and St Vincent's Hospital, University of Melbourne, Richmond, VIC, Australia; ^5^Department of Business Administration, Hong Kong Shue Yan University, Hong Kong, China; ^6^The Jockey Club School of Public Health and Primary Care, Chinese University of Hong Kong, Hong Kong, China; ^7^Unit of Psychiatry, Department of Public Health and Medicinal Administration, Institute of Translational Medicine, Faculty of Health Sciences, University of Macau, Macao, China; ^8^Centre for Cognitive and Brain Sciences, University of Macau, Macao, China; ^9^Institute of Advanced Studies in Humanities and Social Sciences, University of Macau, Macao, China

**Keywords:** older adults, depression, quality of life, high-altitude area, nursing homes

## Abstract

**Objective:** Depressive symptoms (depression hereafter) is common in older adults, and closely associated with environmental factors. This study compared the prevalence of depression in older adults living in high-altitude and low-altitude regions, and their association with quality of life (QOL).

**Method:** A total of 632 older nursing home residents were included, with 425 participants living in low-altitude and 207 participants living in high-altitude regions. Depression and QOL were assessed using standardized instruments.

**Results:** The prevalence of depression was 26.9% (95% CI: 23.43–30.37%) in the whole sample of older nursing home residents, with 11.1% (95% CI: 8.01–14.05%) in those living in low-altitude and 59.4% (95% CI: 52.68–66.17%) in those living in high-altitude regions. Multiple logistic regression analysis revealed that living in low-altitude region (*P* < 0.001, OR = 0.07, 95% CI: 0.04–0.12) was associated with lower risk of depression, while perception of poor health status (*P* < 0.001, OR = 3.86, 95% CI: 1.98–7.54) and having insomnia (*P* < 0.001, OR = 4.76, 95% CI: 2.99–7.56) were associated with higher risk of depression. QOL was significantly lower in physical (*F*_(1,632)_ = 35.421, *P* < 0.001), psychological (*F*_(1,632)_ = 20.777, *P* < 0.001), social (*F*_(1,632)_ = 8.169, *P* < 0.001) and environmental domains (*F*_(1,632)_ = 11.861, *P* < 0.001) in those with depression.

**Conclusion:** Depression was common in older nursing home residents especially those living in the high-altitude region. Considering the negative impact of depression on QOL and functional outcomes, routine screening and timely treatment of depression should be implemented in this population.

## Introduction

Due to biological and psychosocial risk factors such as physical disorders, retirement, bereavement and social isolation, depressive symptoms (depression hereafter) are common in older adults (1, 2), which could lead to a range of negative health outcomes including poor daily functioning, cognitive decline and high risk of suicidality (3–5). The epidemiology of the depression in older adults has been widely studied, with prevalence ranging between 6.35% (6) and 60.3% (7). A meta-analysis revealed that the prevalence of depression was 23.6% among older adults in China (8). The discrepancy in depression prevalence between studies could be partly due to the use of different measures of depression; for example, the Center for Epidemiologic Studies Depression Scale (CES-D) (9), Montgomery–Åsberg Depression Rating Scale (MADRS) (10), Hamilton Rating Scale for Depression (HAMD) (11), and Patients' Health Questionnaire (PHQ-9) (12) were used in different studies.

Environmental factors such as altitude are strongly linked with psychiatric problems (13). For instance, a study conducted in the USA found a positive correlation between increased risk of suicide and high-altitude (14, 15). Living in high-altitude areas is associated with hypoxia (16), lower atmospheric pressure and altered pharmacokinetics of certain psychotropic medications (17), all of which could increase the risk of depression (18). In addition, insufficient health resources, lack of community services, and underdeveloped economic status in many high-altitude areas could be associated with depression (14). However, a common limitation of previous studies of depression in high altitude has been the lack of comparison with those living in low-altitude areas.

Hence, we compared the prevalence of depression in older adults living in low-altitude and high-altitude regions, and explore their association with demographic and clinical characteristics, as well as quality of life (QOL), which is a widely used health outcome in both clinical practice and research (19).

## Methods

### Study Setting and Participants

This cross-sectional comparative study was conducted between September 1^st^ and November 31^st^, 2019 in China. Participants were recruited from three public nursing homes in Xi'ning, a city in Qinghai province with an average altitude of 2,300 m (high-altitude area), and a large-scale nursing home in Guangzhou, a city in Guangdong province, with an average altitude of 10 m (low-altitude area). Older adults in the selected nursing homes were consecutively invited to participate in this study and those who met the following inclusion criteria were included: (1) age of 60 years or above; (2) Chinese ethnicity and fluent in Chinese languages including Mandarin or Cantonese; (3) have ability to communicate and complete the assessment adequately. Those with obvious cognitive decline such as dementia and intellectual disability based on a review of health records were excluded. The study protocol was approved by the IRB of University of the Macau and written informed consent was obtained from all participants.

### Assessment Tools and Evaluation

Basic socio-demographic and clinical characteristics of participants were collected including age, gender, education level, marital status, religion, perceived financial status, medical conditions, and family history of psychiatric disorders. The Chinese version of the self-reported 9-item Patient Health Questionnaire (PHQ-9) was used to evaluate the severity of depression within the past 2 weeks (20, 21). The PHQ-9 is the most commonly used, validated and efficient instrument for screening depression (22), which was developed based on the Diagnostic and Statistical Manual of Mental Disorders-Four Edition (DSM-IV Edition) (23),with satisfactory reliability and validity in Chinese populations including older adults (20). The PHQ-9 consists of nine items, including little interest in doing things, feeling holpless, sleeping disturbance, feeling tired, appetite suiation, feeling bad about self, trouble concentrating on things, moving or speaking slowly, and thought or hurt yourself. Each item is scored from 0 (not at all) to 3 (nearly every day) with a total score between 0 and 27. A higher PHQ-9 total score indicates more severe depression (24), with a total score of ≥5 as “having depression,” and ≥10 as “having moderate to severe depression” (25). Participants' QOL was measured using the World Health Organization Quality of Life brief version (WHOQOL-BREF), which includes 26 items covering four dimensions (physical, psychological, social, and environmental domains). A higher score indicates higher QOL. Following previous studies (26–28), three standardized questions were used to evaluate insomnia symptoms (insomnia hereafter) in the past week (e.g., “Did you have difficulties in initiating sleep?”; “Did you ever have difficulties in maintaining sleep?” and “Did you wake up in the midnight and having difficulties in sleeping again?”), with each question having three options (0 = never, 1 = sometime, and 2 = often). If a participant answered “often” to any of the questions, then he/she was defined as “having insomnia” (28).

### Statistical Analysis

The study data were analyzed using SPSS 24.0 for Windows. Comparison of the basic demographic and clinical characteristics between older adults living in high- and low-altitude regions, and comparison between depression and non-depression groups in the whole sample with respect to socio-demographic and clinical characteristics were performed by independent sample *t*-test, Mann-Whitney *U* test, and chi-square test, as appropriate. The PHQ-9 total scores between low and high altitude areas were compared using analysis of covariance (ANCOVA), after controlling for confounding effects of variables with significant group differences in univariate analyses. In addition, QOL between depression and non-depression groups in the whole sample was also compared using ANCOVA, after controlling for the potentially confounding effects of variables with significant group differences in univariate analyses. Multiple logistic regression analysis with the “enter” method was performed to determine the independent relationships between depression and socio-demographic and clinical characteristics. Depression was entered as dependent variable, while variables that significantly differed between the depression and non-depression groups in the univariate analyses were independent variables. The level of significance was set at 0.05 (two-tailed).

## Result

Of a total of 657 older nursing home residents invited to participate in the study, 632 (425 in low-altitude and 207 in high-altitude regions) met the study criteria and completed the assessments during the study period, giving a participation rate of 96.2%. The prevalence of overall depression (PHQ total score of ≥5) in the whole sample was 26.9% (95% CI: 23.43–30.37%), with 59.4% (95% CI: 52.68–66.17%) in those living in high-altitude and 11.1% (95% CI: 8.01–14.05%) living in low-altitude regions. In contrast, the prevalence of moderate to severe depression (PHQ total score ≥10) was 8.07% (95% CI: 5.94–10.20%) in the whole sample, with 16.91% (95% CI: 11.76–22.06%) living in high-altitude and 3.76% (95% CI: 1.95–5.58%) living in low-altitude areas. The mean total score of PHQ-9 was 3.05 (SD = 3.91).

Table 1 shows the demographic and clinical characteristic of the whole sample and also separately by altitude region. There were significant differences between low-altitude and high-altitude regions in terms of age, ethnicity, education level, religion, perceived financial status, perceived health status, number of medical conditions and depressive symptoms. After controlling for these variables with group differences, older adults living in high altitude area reported more severe depression [*F*_(1,632)_ = 42.849, *P* < 0.001].

**Table 1:** Basic demographic and clinical characteristic of the whole sample.

	**The whole sample** **(*****n*** **=** **632)**		**Low altitude area** **(*****n*** **=** **425)**		**High altitude area** **(*****n*** **=** **207)**		**Statistics**		
	***N***	**%**	***N***	**%**	***N***	**%**	**X^**2**^**	**df^**a**^**	***P***
Male gender	250	39.56	158	37.18	92	44.44	3.08	1	0.079
Ethnicity (Han Chinese)	620	98.10	421	99.06	199	96.14	6.39	1	**0.011**
Married/cohabitating	136	21.52	87	20.47	49	23.67	0.85	1	0.358
Secondary school or above	272	43.04	196	46.12	76	36.71	5.02	1	**0.025**
Smoking	135	21.36	90	21.18	45	21.74	0.03	1	0.871
Having a religion	492	77.85	411	96.71	81	39.13	267.58	1	** <0.001**
Perceived financial status							28.56	2	** <0.001**
Good	256	40.51	144	33.88	112	54.11			
Fair	282	44.62	202	47.53	80	38.65			
Poor	94	14.87	79	18.59	15	7.25			
Family history of psychiatric disorders	14	2.22	9	2.12	5	2.42	0.057	1	0.811
Perceived health status							24.18	2	** <0.001**
Good	152	24.05	82	19.29	70	33.82			
Fair	366	57.91	274	64.47	92	44.44			
Poor	114	18.04	69	16.24	45	21.74			
Any type of insomnia^d^	167	26.42	81	19.06	86	41.54	36.21	1	** <0.001**
	**Mean**	**SD**	**Mean**	**SD**	**Mean**	**SD**	**T/Z**	**df**^**b**^	***P***
Age (year)	80.20	8.50	81.22	8.17	78.12	8.80	4.25	630	** <0.001**
PHQ-9 total	3.05	3.91	1.60	2.89	6.02	4.07	−13.99	–^c^	** <0.001**

a *χ^2^ text;*

b *Two sample independent t-text;*

c *Mann-Whitney U test; Bolded values: p < 0.05; PHQ-9=Patient Health Questionnaire-9;*

d *Major medical conditions included hypertension, cardiopathy, stroke, Parkinson, diabetes, asthma, chronic bronchitis emphysema, pulmonary disease, liver disease, nephropathy, thyroid dysfunction, arthritis, cancer, fracture/osteoporosis/humpback, and other physical diseases. No collinearity was found between variables with significant group differences*.

Table 2 presents the comparisons between older adults with depression and those without depression with respect to basic demographic and clinical characteristics, and QOL. Compared to those without depression, participants with depression had lower education level, no religion, poor health status, were living in higher-altitude region and more likely to have major medical conditions. After controlling for covariates, QOL was significantly lower in physical (*F*_(1,632)_ =35.421, *P* < 0.001), psychological (*F*_(1,632)_ = 20.777, *P* < 0.001), social (*F*_(1,632)_ = 8.169, *P* < 0.001) and environmental (*F*_(1,632)_ = 11.861, *P* < 0.001) domains in those with depression. Multiple logistic regression analysis revealed that living in low-altitude region (*P* < 0.001, OR = 0.07, 95% CI: 0.04–0.12) was associated with lower risk of depression, while perceived poor health status (*P* < 0.001, OR = 3.86, 95% CI: 1.98–7.54) and having insomnia (*P* < 0.001, OR = 4.76, 95% CI: 2.99–7.56) were associated with higher risk of depression (Table 3).

**Table 2:** Comparison between older adults with and without depression in terms of basic demographic and clinical characteristics in the whole sample.

	**Without depression (*****n*** **=** **462)**		**With depression (*****n*** **=** **170)**		**Statistics**		
	***N***	**%**	***N***	**%**	**χ**^**2**^	**df^**a**^**	***P***
Male gender	188	40.69	62	36.47	0.927	1	0.336
Ethnic (Han Chinese)	456	98.70	164	96.47	3.320	1	0.068
Married/cohabitating	96	20.78	40	23.53	0.557	1	0.456
Secondary school or above	210	45.45	62	36.47	4.091	1	**0.043**
Smoking	104	22.51	31	18.24	1.352	1	0.245
Having a religion	395	85.50	97	57.06	58.28	1	** <0.001**
Perceived financial status					3.226	2	0.199
Good	179	38.74	77	45.29			
Fair	216	46.75	66	38.82			
Poor	67	14.50	27	15.88			
Family history of psychiatric disorders	11	2.44	3	1.80	0.218	1	0.641
Perceived health status					23.30	2	** <0.001**
Good	113	24.46	39	22.94			
Fair	286	61.90	80	47.06			
Poor	63	13.64	51	30.00			
Living in low altitude areas	378	81.82	47	27.65	165.57	1	** <0.001**
Any type of insomnia	76	16.45	91	53.53	87.88	1	** <0.001**
	**Mean**	**SD**	**Mean**	**SD**	**T/Z**	**df**^**b**^	***P***
Age(year)	80.53	8.57	79.31	8.26	1.600	630	0.110
Physical QOL	13.43	1.82	12.36	2.25	6.159	630	** <0.001**
Psychological QOL	13.90	1.78	12.43	2.39	8.342	630	** <0.001**
Social QOL	13.59	1.67	12.94	2.25	3.921	630	** <0.001**
Environmental QOL	13.58	1.80	12.80	2.16	4.586	630	** <0.001**

a *χ2 text;*

b *Two sample independent t-text; Bolded values: p < 0.05; PHQ-9=Patient Health Questionnaire-9; QOL= quality of life*.

**Table 3:** Socio-demographic correlates of depression in older adults (by multiple logistic regression analysis).

	**Having depression**		
	**OR**	**95% CI**	***P***
Education	O.73	0.46–1.15	0.18
Having a religion	1.54	0.85–2.80	0.16
Living in low altitude region	0.07	0.04–0.12	** <0.001**
Perceived health status (with “Good” as the reference)	—	—	**—**
Moderate	1.59	0.91–2.76	0.10
Poor	3.86	1.98–7.54	** <0.001**
Any type of insomnia	4.76	2.99–7.56	** <0.001**

*Bolded values: p < 0.05*.

## Discussion

To the best of our knowledge, this was the first study that compared the prevalence of depression in older nursing home residents living in low-altitude and high-altitude regions. Depression was common (26.9%) in older nursing home residents in China, which is similar to the findings in Brazil (23.5%) (29), but higher than those living in nursing homes in Japan (11.9%) (30) and Thailand (18.5%) (31). However, it should be noted that due to different sampling methods, demographic and clinical characteristics, and measures of depression as well as their cutoff values, direct comparisons should be made with caution (8).

The prevalence of depression in older adults living in high-altitude (59.4%) was almost five times higher compared to those living in low-altitude regions (11.1%) in this study, which is consistent with the findings (52.3%) of a previous study conducted in Yushu, another city in Qinghai province (32, 33). The significant difference in depression prevalence between high- and low-altitude regions remained even after controlling for covariates. This could be due to socioeconomic reasons. Generally, the high-altitude region in Qinghai province is one of the most economically under-developed regions in China (34). Poor social welfare, low pension after retirement, and insufficient primary health services in the community are factors associated with an increase the risk of depression in older adults (32, 35). In contrast, Guangzhou (situated in the low-altitude region) is one of the most economically developed areas, with the best social and health service systems in China (34, 36). Second, environmental factors in high-altitude areas, such as hypoxia and lower atmospheric pressure can commonly lead to sleep problems like high apnea/hypopnea index, increased frequency of arousals and changed sleep structure (37–39). In addition, long-term hypoxia in high-altitude areas could influence neural activity through certain neurotransmitters such as dopamine and serotonin in the monoamine system, which in turn could increase the risk of psychiatric problems (32). These factors are also associated with higher risk of depression (40, 41).

In this study apart from living in high-attitude region, perceived poor health status and insomnia were independently associated with higher risk of depression, which is consistent with previous findings (42, 43). Older adults with poor health status particularly severe medical disorders often suffer from physical and psychological problems such as low energy, low appetite, frustration, which may lead to depression. The association between insomnia and depression is bidirectional. On the one hand, common sleep problems including insomnia associated with hypoxia in high-altitude areas can increase the likelihood of depression. On the other hand, depression can also lead to sleep disturbances (43, 44).

Older adults with depression frequently experienced physical distress, impaired functional ability, cognitive decline and higher risk of suicidality (3–5). QOL is deemed to be related to the interaction between protective factors (e.g., better social support) and distressing factors (e.g., poor mental and physical status) (45), hence depressed older adults are likely to have a lower QOL, which is consistent with the our study that QOL was significantly lower in physical, psychological, social and environmental domains in those with depression, although the group differences in QOL ratings were not large.

The strengths of this study were the inclusion of study participants from both high- and low- altitude regions as well as the use of QOL measures. However, several limitations should be noted. First, due to the cross-sectional study design, the causality between depression and other variables could not be examined. Second, as only one public nursing home in low-altitude (Guangzhou) and three public nursing homes in high-altitude regions (Qinghai) were included, the findings could not be generalized to those living in other settings. Finally, older adults with severe cognitive impairment were excluded, which could lead to selection bias to an uncertain extent.

In conclusion, depression was common in older nursing home residents particularly those living in high-altitude region. Considering the negative impact of depression on QOL and functional outcomes, routine screening and timely treatment of depression should be implemented in this subpopulation. In view of the rapid urbanization in China, traditional family structures have gradually changed. Many young family members do not live with their parents/grandparents (46, 47), which results in increasing number of older adults being placed in nursing homes. Therefore, increased mental health care resources should be allocated by the central and local governments to the nursing homes and relevant community mental health services in high-altitude regions to address the needs of this growing sub-population.

## Data Availability Statement

The IRB of University of Macau that approved the study prohibits the authors from making the research data set publicly available. Readers and all interested researchers may contact Dr. Yu-Tao Xiang (Email address: xyutly@gmail.com) for details. Dr. Yu-Tao Xiang could apply to the IRB of University of Macau for the release of the data.

## Ethics Statement

The study protocol was approved by the IRB of University of the Macau and written informed consent was obtained from all participants.

## Author Contributions

Y-TX: study design. FW, SL, QZ, XC, and DZ: collection, analyses, and interpretation of data. FW and Y-TX: drafting of the manuscript. CN: critical revision of the manuscript. All the authors approved the final version for publication.

## Conflict of Interest

The authors declare that the research was conducted in the absence of any commercial or financial relationships that could be construed as a potential conflict of interest.
